# Supplementary figures and images for: Safety of laparoscopic pancreaticoduodenectomy in patients with liver cirrhosis using propensity score matching
Source: PLoS One. 2021 Jan 29;16(1):e0246364. doi: 10.1371/journal.pone.0246364 (PMC7845952; doi:10.1371/journal.pone.0246364)

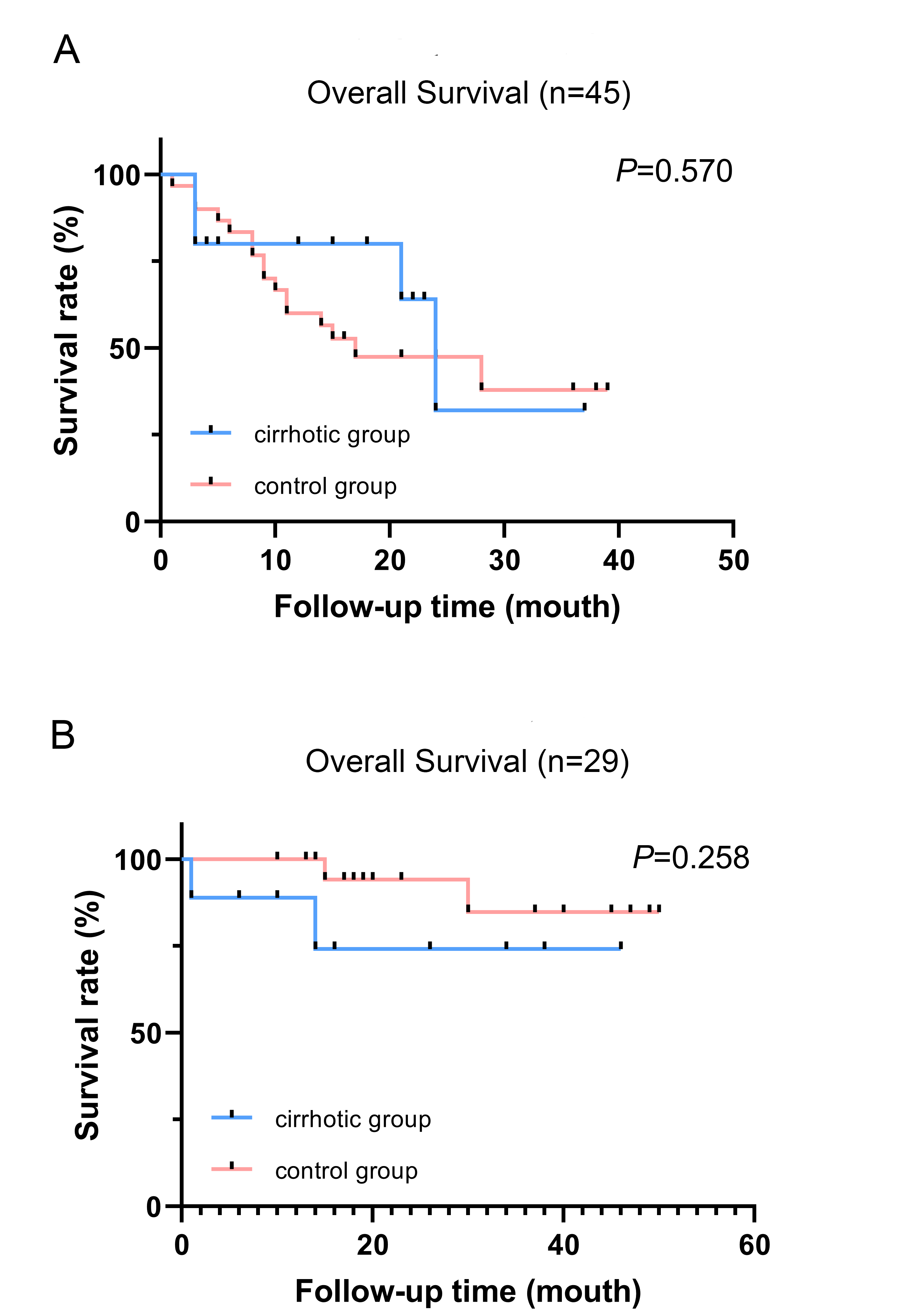

Supplement: S1 Fig — A: Overall survival of cirrhotic patients and control patients with pancreatic carcinoma. B: Overall survival of cirrhotic patients and control patients with periampullary carcinoma. (TIF) [file pone.0246364.s001.tif]
