# Supplementary material for: Safety of laparoscopic pancreaticoduodenectomy in patients with liver cirrhosis using propensity score matching
Source: PLoS One. 2021 Jan 29;16(1):e0246364. doi: 10.1371/journal.pone.0246364 (PMC7845952; doi:10.1371/journal.pone.0246364)
Supplement: S1 Table — (DOCX) [file pone.0246364.s002.docx]

|  | Patients with liver cirrhosis | Control patients | P-value |
| --- | --- | --- | --- |
| **Tumor size (cm)** |  |  |  |
| Pancreatic carcinoma (n=45) | 3.18±1.06 | 3.15±0.99 | 0.926 |
| Periampullary carcinoma （n=29） | 1.95±0.85 | 2.32±1.05 | 0.372 |

**S1 Table. Tumor size according to different tumor between two groups.**
