# Supplementary material for: Safety of laparoscopic pancreaticoduodenectomy in patients with liver cirrhosis using propensity score matching
Source: PLoS One. 2021 Jan 29;16(1):e0246364. doi: 10.1371/journal.pone.0246364 (PMC7845952; doi:10.1371/journal.pone.0246364)

PLOS ONE

Safety of laparoscopic pancreaticoduodenectomy in patients with liver cirrhosis: a propensity score matching study

--Manuscript Draft--

**Manuscript Number:**	PONE-D-20-23981
**Article Type:**	Research Article
**Full Title:**	Safety of laparoscopic pancreaticoduodenectomy in patients with liver cirrhosis: a propensity score matching study
**Short Title:**	laparoscopic pancreaticoduodenectomy and liver cirrhosis
**Corresponding Author:**	Bing Peng<br>Sichuan University West China Hospital<br>Chengdu, China CHINA
**Keywords:**	laparoscopic pancreaticoduodenectomy; liver cirrhosis; postoperative complications; postoperative mortality
**Abstract:**	To date, the safety of laparoscopic pancreaticoduodenectomy (LPD) in patients with liver cirrhosis is still unclear. The study aims to report the postoperative morbidity and mortality and long-terms result of cirrhotic patients underwent LPD. Retrospective ly, one center study of 353 patients underwent LPD between October 2010 and December 2019. Only 28 patients had liver cirrhosis and paired with 56 non-cirrhotic counterparts by propensity score matching (PSM). Baseline data, intra-operative data, postoperative data, and survival data were collected. The primary result is postoperative morbidity, second results including postoperative mortality, surgical parameters, and long-term overall survival.<br>The cirrhotic patients and control patients respectively had postoperative complication rates of 82% and 48% (P=0.003), Clavien-Dindo ≥III complication rates of 14% and 11% (P=0.634), Clavien-Dindo I-II complication rates of 68% and 38% (P=0.009), hospital mortality of 4% and 2% (P=0.613), the overall survival of 32 months and 34.5 months (P=0.991), intraoperative blood loss of 300 (200-400) ml and 150 (100-250) ml (P<0.0001), drain amount of 2572.5 (1023.8-5275) ml and 1617.5 (907.5-2700) ml (P=0.048). In conclusion, LPD is associated with an increased risk of postoperative morbidity in patients with liver cirrhosis. However, the incidence of Clavien-Dindo ≥III complications and post-operative mortality did not increase, and liver cirrhosis was no association for worse overall survival in patients who underwent LPD. It indicates t hat patients with liver cirrhosis could routinely be considered for LPD at high volume centers with rigorous selection and management.
**Order of Authors:**	Ke Cheng
	Wei Liu
	Jiaying You
	Shashi Shah
	Yunqiang Cai
	Xin Wang
	Bing Peng
**Additional Information:**	
**Question**	**Response**
**Financial Disclosure**<br><br>Enter a financial disclosure statement that describes the sources of funding for the work included in this submission. Review the submission guidelines for detailed	The author (s) received no specific funding for this work.

Powered by Editorial Manager® and ProduXion Manager® from Aries Systems Corporation

requirements. View published research articles from *PLOS ONE* for specific examples.<br><br>This statement is required for submission and **will appear in the published article** if the submission is accepted. Please make sure it is accurate.<br><br>**Unfunded studies**<br>Enter: *The author(s) received no specific funding for this work.*<br><br>**Funded studies**<br>Enter a statement with the following details:<br>• Initials of the authors who received each award<br>• Grant numbers awarded to each author<br>• The full name of each funder<br>• URL of each funder website<br>• Did the sponsors or funders play any role in the study design, data collection and analysis, decision to publish, or preparation of the manuscript?<br>• **NO** - Include this sentence at the end of your statement: *The funders had no role in study design, data collection and analysis, decision to publish, or preparation of the manuscript.*<br>• **YES** - Specify the role(s) played.<br><br>* typeset	
**Competing Interests**<br><br>Use the instructions below to enter a competing interest statement for this submission. On behalf of all authors, disclose any competing interests that could be perceived to bias this work—acknowledging all financial support and any other relevant financial or non-financial competing interests.<br><br>This statement **will appear in the published article** if the submission is accepted. Please make sure it is accurate. View published research articles from *PLOS ONE* for specific examples.	The authors have declared that no competing interests exist.

**NO authors have competing interests**<br><br>Enter: *The authors have declared that no competing interests exist.*<br><br>**Authors with competing interests**<br><br>Enter competing interest details beginning with this statement:<br><br>*I have read the journal's policy and the authors of this manuscript have the following competing interests: [insert competing interests here]*<br><br>* typeset	
**Ethics Statement**<br><br>Enter an ethics statement for this submission. This statement is required if the study involved:<br><br>• Human participants<br>• Human specimens or tissue<br>• Vertebrate animals or cephalopods<br>• Vertebrate embryos or tissues<br>• Field research<br><br>Write "N/A" if the submission does not require an ethics statement.<br><br>General guidance is provided below. Consult the submission guidelines for detailed instructions. **Make sure that all information entered here is included in the Methods section of the manuscript.**	The study was approved by the ethics committee of Sichuan University in 2018. The approval number was 2018年 审（97）号. The data were analyzed anonymously.

Powered by Editorial Manager® and ProduXion Manager® from Aries Systems Corporation

**Format for specific study types**<br><br>**Human Subject Research (involving human participants and/or tissue)**<br>• Give the name of the institutional review board or ethics committee that approved the study<br>• Include the approval number and/or a statement indicating approval of this research<br>• Indicate the form of consent obtained (written/oral) or the reason that consent was not obtained (e.g. the data were analyzed anonymously)<br><br>**Animal Research (involving vertebrate animals, embryos or tissues)**<br>• Provide the name of the Institutional Animal Care and Use Committee (IACUC) or other relevant ethics board that reviewed the study protocol, and indicate whether they approved this research or granted a formal waiver of ethical approval<br>• Include an approval number if one was obtained<br>• If the study involved *non-human primates*, add *additional details* about animal welfare and steps taken to ameliorate suffering<br>• If anesthesia, euthanasia, or any kind of animal sacrifice is part of the study, include briefly which substances and/or methods were applied<br><br>**Field Research**<br><br>Include the following details if this study involves the collection of plant, animal, or other materials from a natural setting:<br>• Field permit number<br>• Name of the institution or relevant body that granted permission	
**Data Availability**<br><br>Authors are required to make all data underlying the findings described fully available, without restriction, and from the time of publication. PLOS allows rare exceptions to address legal and ethical concerns. See the PLOS Data Policy and FAQ for detailed information.	Yes - all data are fully available without restriction

A Data Availability Statement describing where the data can be found is required at submission. Your answers to this question constitute the Data Availability Statement and **will be published in the article**, if accepted.<br><br>**Important:** Stating 'data available on request from the author' is not sufficient. If your data are only available upon request, select 'No' for the first question and explain your exceptional situation in the text box.<br><br>Do the authors confirm that all data underlying the findings described in their manuscript are fully available without restriction?	
**Describe where the data may be found in full sentences. If you are copying our sample text, replace any instances of XXX with the appropriate details.**<br><br>• If the data are **held or will be held in a public repository**, include URLs, accession numbers or DOIs. If this information will only be available after acceptance, indicate this by ticking the box below. For example: *All XXX files are available from the XXX database (accession number(s) XXX, XXX.).*<br>• If the data are all contained **within the manuscript and/or Supporting Information files**, enter the following: *All relevant data are within the manuscript and its Supporting Information files.*<br>• If neither of these applies but you are able to provide **details of access elsewhere**, with or without limitations, please do so. For example:<br><br>*Data cannot be shared publicly because of [XXX]. Data are available from the XXX Institutional Data Access / Ethics Committee (contact via XXX) for researchers who meet the criteria for access to confidential data.*<br><br>*The data underlying the results presented in the study are available from (include the name of the third party*	All relevant data are within the manuscript

and contact information or URL).<br>• This text is appropriate if the data are owned by a third party and authors do not have permission to share the data.<br><br>* typeset	
Additional data availability information:	Tick here if the URLs/accession numbers/DOIs will be available only after acceptance of the manuscript for publication so that we can ensure their inclusion before publication.

Powered by Editorial Manager® and ProduXion Manager® from Aries Systems Corporation

Manuscript
Click here to access/download;Manuscript;manuscript.docx

Safety of laparoscopic pancreaticoduodenectomy in patients with liver cirrhosis: a propensity score matching study

Ke Cheng[1], Wei Liu[1], Jiaying You[1], Shashi Shah[1], Yunqiang Cai[1], Xin Wang[1], Bing Peng[1*]

[1]The West China Hospital, Sichuan University, Chengdu, Sichuan, China

[*]Corresponding author

Email: drpengbing123@163.com (BP)

Abstracts

To date, the safety of laparoscopic pancreaticoduodenectomy (LPD) in patients with liver cirrhosis is still unclear. The study aims to report the postoperative morbidity and mortality and long-terms result of cirrhotic patients underwent LPD. Retrospectively, one center study of 353 patients underwent LPD between October 2010 and December 2019. Only 28 patients had liver cirrhosis and paired with 56 non-cirrhotic counterparts by propensity score matching (PSM). Baseline data, intra-operative data, postoperative data, and survival data were collected. The primary result is postoperative morbidity, second results including postoperative mortality, surgical parameters, and long-term overall survival. The cirrhotic patients and control patients respectively had postoperative complication rates of 82% and 48% (P=0.003), Clavien-Dindo ≥III complication rates of 14% and 11% (P=0.634), Clavien-Dindo I-II complication rates of 68% and 38% (P=0.009), hospital mortality of 4% and 2% (P=0.613), the overall survival of 32 months and 34.5 months (P=0.991), intraoperative blood loss of 300 (200-400) ml and 150 (100-250) ml (P<0.0001), drain amount of 2572.5（1023.8-5275）ml and 1617.5（907.5-2700） ml (P=0.048). In conclusion, LPD is associated with an increased risk of postoperative morbidity in patients with liver cirrhosis. However, the incidence of Clavien-Dindo ≥III complications and post-operative mortality did not increase, and liver cirrhosis was no association for worse overall survival in patients who underwent LPD. It indicates that patients with liver cirrhosis could routinely be considered for LPD at high volume centers with rigorous selection and management.

Keywords: laparoscopic pancreaticoduodenectomy; liver cirrhosis; postoperative complications; postoperative mortality

Introduction

Pancreaticoduodenectomy (PD) is the main surgical option for managing pancreatic and, duodenal neoplasms as well as other lesions located in the pancreatic head and periampullary region [1]. On the other hand, laparoscopic pancreaticoduodenctomy (LPD) is one of the most challenging gastrointestinal operations. This approach is identical to PD, while LPD results in short hospital stays, low blood loss, fewer complications, and long-term overall survival outcomes [2, 3]. However, LPD is not widely accepted as a routine operation, owing to its technical limitations [4, 5].

Liver cirrhosis is a life-threatening health problem that results in multiple serious complications, including variceal bleeding, ascites, spontaneous bacterial peritonitis, and encephalopathy [6]. In most parts of Asia, Hepatitis B virus (HBV) infection represents the leading cause of liver cirrhosis [6], with China also ranked high due to correspondingly high HBV incidence. The cirrhotic patients are prone to increased risk of bleeding, infections, hepatic decompensation, and hepatic coma following a major abdominal operation [6]. Liver cirrhosis negatively affects outcomes after intra-abdominal surgery [7], with various studies reporting high postoperative morbidities and mortalities in cirrhotic patients [8, 9]. For example, Nguyen *et al* [9] reported postoperative complications were more likely in cirrhotic patients (adjusted odds ratio, 1.35; 95% confidence interval, 1.20-1.52) relative to patients with no cirrhosis following colorectal surgery. Besides, patients with cirrhosis had significantly higher in-hospital mortality than patients with no cirrhosis (14% vs. 5%, respectively, $P < 0.0001$). In the past, the disease was considered a contraindication for pancreatic or biliary tract surgery [10]. Currently, a significant improvement in surgical techniques and medical management has led to the development of various surgical procedures for safe application in cirrhotic patients [11, 12]. Numerous studies have

reported the application of laparoscopic surgery, as a safe and less invasive alternative to open surgery, in cirrhotic patients [13, 14]. For example, Kim *et al* [15] reported that laparoscopic gastrectomy (LG) in gastric cancer patients with liver cirrhosis was safe. Also, Zhou *et al* [13] reported that laparoscopic colorectal surgery is a safe and less invasive alternative for open surgery in some elective cirrhotic patients.

To date, the safety of LPD in cirrhotic patients remains unknown, although a few studies have described outcomes of PD in patients with liver cirrhosis [16-19]. Considering the difference between PD and LPD, it is necessary to study the short and long outcomes of LPD in patients with liver cirrhosis. The aim of this retrospective cohort study was, therefore, to evaluate the effect of liver cirrhosis on postoperative morbidity, mortality, and overall survival outcomes in patients who underwent LPD in our center. To find whether LPD is a safe procedure for cirrhotic patients.

Materials and methods

Study design

We retrospectively evaluated all patients who underwent LPD at the Pancreatic department, West China Hospital, between October 2010 and December 2019. All data, which were collected in May 2020, were fully anonymized before we accessed them. We could not identify individual participants during or after data collection. Patients diagnosed with resectable malignant tumors or benign diseases located in the periampullary or pancreas, who underwent LPD, were included in the study. Exclusion criteria comprised patients who were postoperatively treated in other medical centers or converted to laparotomy, patients with poor liver function, and patients with thrombosed portal veins, malnutrition, and severe malignant tumors. Diagnosis of cirrhosis was confirmed via ultrasound, CT, and intra-operative findings or Liver biopsies.

We reviewed medical records for all the patients then paired each cirrhotic patient with two non-cirrhotic counterparts using the PSM method [20] to minimize the influence of other confounders such as age, gender, body mass index (BMI), and diagnosis of diseases. The primary result is post-operative morbidity, second results including mortality, surgical parameters, and long-term overall survival. The study was approved by the ethics committee of Sichuan University.

Preoperative assessment

We collected demographic data for each patient, including their age, gender, BMI, diagnosis, American society of anesthesiologists classification (ASA) [21], and laboratory tests (blood tests, liver and kidney function tests, coagulation function tests). Additionally, we graded liver function for the cirrhotic patients according to the Child-Pugh classification [22] and the Model for End-stage Liver Disease (MELD) score [23, 24], then performed a revaluation of Child-Pugh class after biliary drainage.

Operative data

All LPD operations were performed by the same group of surgeons using standard procedures which was described in a previous publication [25]. Operative information was collected, including operative time, blood loss, blood transfusion, pancreatic texture and duct diameter, site of the tumor, tumor size, and vascular resection.

Postoperative data

Summarily, the drain was removed from all patients in cases where no bile, pancreatic, or other leaks were present. Postoperative laboratory tests were measured on 3 postoperative days (POD). Thereafter, the hospital, and 90-day mortalities, the incidence of reoperation, the period of ICU, and hospital stay, drain removed and the amount, as well as postoperative complications, were recorded.

Definitions

All postoperative complications were stratified using the Clavien-Dindo classification of surgical complications [26]. Postoperative pancreatic fistula (POPF), delayed gastric emptying (DGE), post-pancreatectomy hemorrhage bleeding (PPH) were defined according to the International Study Group of Pancreatic Fistula (ISGPF) [27-29], whereas postoperative ascites was defined as the effusion of more than 400ml/d through the drain after POD 4 [30]. Reoperation was defined as a secondary operation because of severe complications following LPD, whereas hospital mortality was defined as death within 30 days following surgery or during primary hospitalization.

Follow-up

For the first 2 years after LPD, follow-up examinations (blood tests, liver and kidney function tests, abdominal CT scans) were conducted at 3-months intervals. If patients exhibited no signs of recurrence, 2 years after surgery, follow-up examinations were changed to a 6-months interval. The follow-up data were collected by examining outpatient medical records as well as personal communication by telephone.

Statistical analysis

PSM was calculated using logistic regression to minimize the potential bias between two groups, and a 2:1 patient matching was performed using the nearest-neighbor matching method without replacement. A caliper radius equal to a standard deviation of 0.1 was set to prevent poor matching. The variables included in the matching model were gender, age, BMI, and diagnosis.

For continuous variables, results were reported as means ± standard deviations of the mean (SD) for normally-distributed data. Otherwise, the median (25 quantile-75 quantiles) was reported. For categorical variables, frequencies and percentages were reported. Categorical variables were assessed using the Chi-square test. Continuous variables were analyzed using the Student *t*-test for normally

distributed data. Otherwise, the non-parametric test was used. The Kaplan-Meier method [31] was adopted for calculating the overall survival, using GraphPad Prism (version 8, GraphPad Software, San Diego, USA), and the results compared by the log-rank test. Values with P <0.05 were considered statistically significant by the two-tailed test. All statistical analyses were performed using the Statistical Product and Service Solutions (SPSS) statistical software (version 26, IBM Corporation, New York, USA).

Results

Patient characteristics

A total of 353 patients met the aforementioned inclusion criteria. Before the PSM, the diagnosis of pancreatic carcinoma was significantly different between the two cohort groups (P=0.03). After the PSM, 28 patients in the cirrhotic group and 56 patients in the control group composed the matched cohort, and bias factors were modified (Table 1). All results of the study were based on PSM analysis. In the cirrhotic group, 15 (54%) patients were diagnosed with pancreatic carcinoma, 3 (11%) had chronic pancreatitis, 9 (32%) exhibited periampullary carcinoma whereas 1 (4%) had other benign diseases. Besides, 11 (39%) patients had Child-Pugh A liver function, 17 (61%) had Child-Pugh B liver function and none was found with Child-Pugh C liver function. The median MELD score was 12 (8-17). We found no statistically significant differences in their baseline conditions between the cirrhotic and control groups. Specifically, in the cirrhotic group, 16 (57%) patients were ASA II, whereas 12 (43%) were ASA III albeit with no significant differences between the two groups (P=0.19). Preoperative laboratory examinations revealed no significant differences between the two groups. All preoperative results are summarized in Table 2.

Table 1. Comparison of baseline characteristics between the two groups before and after PSM.

	Before PSM			After PSM		
Characteristic	Cirrhosis(n=28)	Control(n=325)	P-value	Cirrhosis(n=28)	Control(n=56)	P-value
**Gender**			0.098			1
Male	21[75]	192[59]		21[75]	42[75]	
Female	7[25]	133[41]		7[25]	14[25]	
**Age (years)**	62 (50-70.5)	62 (51-70)	0.995	62 (50-70.5)	60 (52-71)	0.798
**BMI (kg/m^2)**	21.4±2.1	21.7±2.9	0.635	21.4±2.1	21.5±1.7	0.686
**Diagnosis**						
Pancreatic carcinoma	15[54]	108[33]	**0.03**	15[54]	30[54]	1
Periampullary carcinoma	9[32]	133[41]	0.363	9[32]	20[36]	0.746
Benign diseases	4[14]	84[26]	0.175	4[14]	6[11]	0.905

BMI: Body mass index, PSM: Propensity score matching.

Table 2. Baseline of patients between cirrhotic and control groups.

	Patients with liver cirrhosis [%]	Control patients [%]	P-value
**Patients**	28	56	
**Gender**			1
Male	21[75]	42[75]	
Female	7[25]	14[25]	
**Age (years)**	62 (50-70.5)	60 (52-71)	0.798
**BMI (kg/m^2)**	21.4±2.1	21.5±1.7	0.686
**Diagnosis**			
Pancreatic carcinoma	15[54]	30[54]	1
Chronic pancreatitis	3[11]	2[4]	0.415
Periampullary carcinoma	9[32]	20[36]	0.746
Other benign diseases	1[4]	4[7]	0.87
**ASA score**			0.19
II	16[57]	40[71]	
III	12[43]	16[29]	
**Cause of liver cirrhosis**			
HBV	13[46]		
Alcohol abuse	4[14]		
Biliary-oriental cirrhosis	7[25]		
Schistosomiasis	1[4]		
Cryptogenic cirrhosis	3[11]		
**Child-Pugh classification**			
A	11[39]		
B	17[61]		
**MELD score**	12(8-17)		

**Preoperative laboratory Examinations**			
Blood platelet (10^9)	188 (133.3-278)	206 (147.3-261.8)	0.491
RBC (10^{12})	3.9±0.6	4.1±0.5	0.125
WBC (10^{12})	5.9 (3.9-8.1)	5 (4-6.6)	0.273
Albumin(g/L)	37.3±5.4	38.9±5.7	0.205
Total bilirubin(μmol/L)	93.6（14.9-186.5）	77.7（19.8-208.9）	0.824
Direct bilirubin (μmol/L)	84.6（5.4-172.6）	69.3（9.4-191.1）	0.827
ALT (g/L)	88（24.8-212.3）	78.5（25.4-150）	0.462
AST (g/L)	86（26-111.8）	66（23-156.8）	0.876
Serum creatinine (μmol/L)	67.5（62-78.5）	72（62.5-81.8）	0.217
PT(s)	11.9（11-12.7）	11.8（11.2-12.9）	0.831
INR	1.04（0.95-1.11）	1.03（0.97-1.11）	0.754

BMI: Body mass index, ASA: American society of anesthesiologists, HBV: Hepatitis B virus, MELD: Model for End-stage Liver Disease, ALT: Alanine transaminase, AST: Aspartate aminotransferase, PT: Prothrombin time, INR: International normalized ratio, RBC: Red blood cell, WBC: White blood cell.

Operative data

We found no statistically significant differences between the groups in terms of tumor size, pancreatic texture, and duct diameter, as well as operative time. However, a significantly lower median intraoperative blood loss of 150 (100-250) ml was recorded in the control group, relative to 300 (200-400) ml in the cirrhotic group (P<0.0001). No significant differences were also detected in the proportion of patients receiving an intraoperative transfusion. Results from all operative evaluations are summarized in Table 3.

Table 3. Operative data and characteristics of patients between cirrhotic and control groups.

	Patients with liver cirrhosis [%] (n=28)	Control patients [%] (n=56)	P-value
**Operative time (min)**	380.7±84.4	352.3±93.3	0.173
**Blood loss (ml)**	300（200-400）	150（100-250）	**＜0.0001**
**Blood transfusion**	2[7]	3[5]	0.744
**Pancreatic texture**			0.636
Soft	10[36]	23[41]	
Firm	18[64]	33[59]	
**Pancreatic duct diameter (mm)**	4(3-5.5)	3(3-4)	0.127
**Site of tumor**	25	54	
Ampulla	0[0]	4[7]	0.398
Duodenum	7[28]	10[19]	0.34

Common bile	2[8]	6[11]	0.98
Pancreas	16[64]	34[63]	0.929
**Tumor size (cm)**	2.5 (1.6-3.4)	3 (2.2-4.5)	0.175
**Vascular resection**	5[18]	13[23]	0.573

Postoperative data

Results revealed similar overall hospital (P=0.613) and 90-day (P=0.163) mortalities between the groups. Similarly, the reoperation rate showed no significant differences between the two groups (P=0.793). Significantly higher drain amounts were found in the cirrhotic patients relative to the control group, recording 2572.5 (1023.8-5275) ml and 1617.5 (907.5-2700) ml, respectively (P=0.048). However, the drain removal days were similar (P=0.293). Furthermore, there were no significant differences between the groups with regards to ICU and hospital stays. The incidence of overall postoperative complications were 82% and 48% (P=0.003) in the cirrhotic and control groups, respectively. Morbidities of complications in Clavien-Dindo I-II were 68% and 38% (P=0.009) in the cirrhotic and control groups, respectively, whereas the respective severe complication rates in Clavien-Dindo ≥III were 14% and 11% (P=0.634) for the two groups. We also found the cirrhotic group had a higher pulmonary infection rate compared with control groups (P=0.003). The results for the complications are listed in Table 4. A comparison between groups indicated a higher incidence of Clavien-Dindo I-II complications (P=0.016) in Child B than control groups. The results of the Child-Pugh classification are summarized in Table 5.

Table 4. Postoperative outcome of patients between cirrhotic and control groups.

	Patients with liver cirrhosis [%] (n=28)	Control patients [%] (n=56)	P-value
**Hospital mortality**	1[4]	1[2]	0.613
**90-day mortality**	4[14]	3[5]	0.163
**Reoperation**	3[11]	5[9]	0.793
**ICU stay**	25[89]	51[91]	0.793
**Days on ICU**	1（1-2）	1（1-2）	0.58

**Hospital days**	26（23-35）	23.5（20-31.8）	0.114
**Drain remove days**	10.5（7-14.8）	8（7-12）	0.293
**Drain amount (ml)**	2572.5（1023.8-5275）	1617.5（907.5-2700）	**0.048**
**Postoperative complications**	23[82]	27[48]	**0.003**
Clavien-Dindo I-II	19[68]	21[38]	**0.009**
Clavien-Dindo ≥III	4[14]	6[11]	0.634
Pancreatic fistula	8[29]	10[18]	0.259
A	4[14]	6[11]	0.905
B	4[14]	2[4]	0.178
C	0	2[4]	0.55
Delayed gastric emptying	8[27]	9[16]	0.179
Biliary leakage	1[4]	1[2]	0.613
Anastomotic fistula	0	0	
Lymphatic fistula	6[21]	5[9]	0.109
Post-pancreatectomy hemorrhage bleeding	2[7]	1[2]	0.212
Abdominal infection	5[18]	5[9]	0.234
Wound infection	2[7]	2[4]	0.469
Pulmonary infection	10[36]	5[9]	**0.003**
**Postoperative laboratory examinations**			
Blood platelet (10^9)	176（120.8-225.8）	163.5（127.5-239.8）	0.761
Albumin (g/L)	28.7±3.9	28.1±3.8	0.498
Total bilirubin (μmol/L)	46.2（22.2-122）	42.5（22-98.2）	0.928
Direct bilirubin (μmol/L)	42.1（10.2-107.3）	32（12.1-89.1）	0.887
ALT (g/L)	44.5（28.3-88.3）	34（18.8-95）	0.159
AST (g/L)	31.5（20-60.3）	28.5（17.5-81.8）	0.417
Serum creatinine (μmol/L)	64（48.3-72.5）	60（49-74）	0.237
PT (s)	12.7（11.4-13.3）	12.1（11.5-12.9）	0.491
INR	1.09（1.01-1.13）	1.07（1-1.12）	0.54

ICU: intensive care unit, ALT: Alanine transaminase, AST: Aspartate aminotransferase, PT: Prothrombin time, INR: International normalized ratio.

Table 5. Outcomes of cirrhotic patients with Child classification.

	Child A	Control	P-value	Child B	Control	P-value
Patients	11	56		17	56	
**ASA score**			0.454			0.327
II	6[54]	40[71]		10[59]	40[71]	
III	5[42]	16[29]		7[41]	16[29]	
MELD score	7(6-9)			16(13.5-18)		
**Complications related to pancreatic surgery**						
Clavien-Dindo I-II	7[64]	21[38]	0.203	12[71]	21[38]	**0.016**
Clavien-Dindo≥III	1[9]	6[11]	0.87	3[18]	6[11]	0.734
Pancreatic fistula	1[9]	10[18]	0.785	7[41]	10[18]	0.096
Delayed gastric emptying	3[27]	9[16]	0.649	5[29]	9[16]	0.383

Biliary leakage	1[9]	1[2]	0.263	0	1[2]	0.465
Lymphatic fistula	3[27]	5[9]	0.228	3[18]	5[9]	0.572
Intra-abdominal bleeding	2[18]	1[2]	0.068	0	1[2]	0.767
Abdominal infection	1[9]	5[9]	0.675	4[24]	5[9]	0.237
Wound infection	1[9]	2[4]	0.421	1[6]	2[4]	0.554

ASA: American society of anesthesiologists, MELD: Model for End-stage Liver Disease.

In the cirrhotic group, a total of 12 (43%) patients developed postoperative ascites, 4 (14%) had deterioration of hepatic function and 1 (4%) exhibited upper gastrointestinal tract bleeding. According to Child-Pugh classification, postoperative ascites, deterioration of hepatic function, and upper gastrointestinal tract bleeding were not significantly different between Child A and Child B classes. The MELD scores for Child A and Child B groups were 7 (6-9) and 16 (13.5-18) ($P<0.0001$), respectively (Table 6).

Table 6. Comparison of liver cirrhosis relative outcomes of patients between Child A and Child B.

	Child A	Child B	P-value
Patients	11	17	
MELD score	7(6-9)	16(13.5-18)	**＜0.0001**
**Complications related to cirrhosis**			
Ascites	3[27]	9[53]	0.18
Deterioration of hepatic function	2[18]	2[12]	0.636
Upper gastrointestinal tract bleeding	1[9]	0	0.206

MELD: Model for End-stage Liver Disease.

Survival data

Patients with malignant diseases were analyzed by the Kaplan-Meier method. At follow-up, a total of 25 (34%) patients were lost. We found no significant difference (P=0.991) in the overall survival rate between cirrhotic patients (32 months) and the control group (34.5 months) (Fig 1).

Fig 1 Overall survival in cirrhotic patients and control patients (malignant diseases).

Discussion

This study describes the safety of LPD in patients with liver cirrhosis. Our main results reveal that the Clavien-Dindo ≥III complication rates and hospital mortality were no differences between the 2 groups under study. Although liver cirrhosis brings ~~with~~ a higher incidence of overall postoperative complications, patients with pancreatic and periampullary diseases, as well as those with liver cirrhosis could routinely be considered for LPD at high volume centers with rigorous selection and management. LPD is a complicated procedure with possibly less blood loss, a less complication rate, and similar overall survival rates compared with those in PD [3]. However, the procedure can only be routinely applied at high volume centers, and by experienced surgeons, since it requires minimal invasive pancreatic operation skills and the steep learning curve [32]. Currently, the operative time is no longer a drawback for LPD, owing to the improvements made in its operative technique. The approach has subsequently been used, with Delitto *et al* [33] reporting 52 cases of LPD in 2017 at a mean operative time of 361 minutes. In our study, the mean operative time for the control group was 352.3 minutes. Our result suggesting that cirrhosis is not a risk factor for longer operative time. A handful of studies have reported the impact of cirrhosis on the surgical outcome of PD [16-19]. However, there is no study has reported the post-operation morbidity and mortality of patients with liver cirrhosis, who underwent LPD.

Patients with liver cirrhosis have a poor response to surgical stress secondary to loss of liver reserves and because of other systemic derangements that result from hepatic dysfunction. At our center, the incidence of liver cirrhosis patients included in the study, who underwent LPD, is 7.93%. Other studies have reported that between 1.9 and 15.2% of patients receiving PD have liver cirrhosis [16-19]. Laparoscopic techniques can improve their outcomes [34]. For example, EI Nakeeb[19] *et al* reported

that wound complications, pancreatic fistula, and hospital mortality were significantly higher in cirrhotic patients compared to the control group. Similarly, Regimbeau *et al* [18] found a higher postoperative morbidity rate in the cirrhotic relative to the control group (86% and 43%, P<0.001). Also, postoperative mortality rates in cirrhotic and control patients were 17 and 5% (P=0.94), respectively. In the current study, the hospital mortality rates were 4 and 2% (P=0.613) in cirrhotic and control groups, respectively, which was lower than studies on PD. Another study [35], investigating 550 patients at our center, reported the hospital mortality rate of less than 1% of LPD. Minimally-invasive of LPD may contribute to the result. In our study, only one patient in the cirrhotic group died because of postoperative hepatic function failure and pulmonary infection. Besides, we graded complications related to pancreatic surgery using the Clavien-Dindo classification, and there was no significant difference in severe complications (Clavien-Dindo≥III) between the cirrhotic and control groups. These results were different from other related studies on PD [17, 18], indicating that LPD as a minimally-invasive procedure may be safer for cirrhotic patients compared to PD. Patients with chronic liver diseases are at high risk of contracting infections, due to increased bacterial translocation and immune suppression associated with a decrease in liver function. For example, pneumonia is reportedly a common infectious disease in cirrhotic patients [36, 37]. In the current study, we found significantly higher morbidity of postoperative pulmonary infection in cirrhotic than control patients. This indicates that cirrhotic patients should be encouraged to take actions such as coughing, sputum expulsion, and initiation of deep breathing or early application of antibiotics, to reduce the risk of pulmonary infection [38]. We also found significantly higher intraoperative blood loss in cirrhotic patients, relative to the control group, which attributed to bleeding tendency and portal hypertension [34]. For example, EI Nakeeb *et al* [19] reported a median blood loss of 500 ml in the cirrhotic group,

relative to 200 ml in the control group (P=0.0001). In the current study, the amount of postoperative drain in cirrhotic patients was significantly higher than in the control group. Postoperative ascites and lower levels of albumin may result in more drain amount in cirrhotic patients.

We also adopted the Child-Pugh classification and MELD scores for assessing preoperative hepatic function. However, it is important to note that in our study, not each patient with obstructive jaundice was treated with standardized biliary drainage during the long study period, which may have affected the assessment of Child-Pugh classification and the MELD score. Also, we recorded a median MELD score of 12 (8-17), which may be increased artificially in patients with pancreatic disease resulting from increased bile duct obstruction and does not directly reflect liver function disorders. Therefore, we cannot conclude whether critical MELD scores are effective for predicting poor outcomes in patients with LPD, owing to the possibility of overestimated assessment.

According to long-term results from cirrhotic patients who underwent LPD, we found no significant differences in the mean survival rate of cirrhotic patients, relative to the control patients. It indicates that liver cirrhosis may not be an association for poor overall survival outcomes in patients with LPD. The results were similar to those reported by other studies [18, 19], although the inclusion of patients with different diseases in our study, may have affected the estimation of overall survival. The findings of our study also revealed that cirrhotic patients did not have a prolonged hospital and ICU stay times, which was different from other research in cirrhotic patients who underwent PD. For example, Warnick *et al* [17] found that cirrhotic patients stayed longer in the ICU than control patients, whereas EI Nakeeb *et al* [19] reported that cirrhotic patients had a prolonged hospital stay and significantly longer ICU stay than control patients. These findings supported the idea that LPD is a less invasive procedure, with less ICU stay time than PD for patients with liver cirrhosis.

Our study still has some limitations. Firstly, the study employed a retrospective design and had a limited number of cases. Secondly, although patients with liver cirrhosis in Child-Pugh B class safely underwent LPD in our study, caution is advised before drawing concrete conclusions because this is one single-center study and the preoperative arrangement of obstructive jaundice were not fully standardized. Thirdly, portal hypertension is an important complication of liver cirrhosis. However, we fail to analyze it during to a lack of standardized assessment of portal hypertension. Multicenter prospective studies with standardized included criteria are needed to validate our findings.

Conclusion

In conclusion, LPD is associated with an increased risk of postoperative morbidity in patients with liver cirrhosis. However, the incidence of Clavien-Dindo ≥III complications and postoperative mortality did not increase, and liver cirrhosis was no association for worse overall survival in patients who underwent LPD. Patients with pancreatic and periampullary diseases, as well as those with liver cirrhosis could be routinely considered for LPD at high volume centers with rigorous selection and management. LPD could be a more safe approach for managing liver cirrhosis in patients compared with DP.

Acknowledgments

This work was supported by grants from the 1.3.5 project for disciplines of excellence-clinical Research Incubation Project, West China Hospital, Sichuan University (No.2018HXFH015). The authors have no conflicts of interest to declare. The study was approved by the ethics committee of Sichuan University.

References

1. Senda Y, Shimizu Y, Natsume S, Ito S, Komori K, Abe T, et al. Randomized clinical trial of duct-to-mucosa versus invagination pancreaticojejunostomy after pancreatoduodenectomy. Br J Surg.

2018;105(1):48-57. Epub 2017/12/22. doi: 10.1002/bjs.10727. PubMed PMID: 29265404.
2. Liao CH, Wu YT, Liu YY, Wang SY, Kang SC, Yeh CN, et al. Systemic Review of the Feasibility and Advantage of Minimally Invasive Pancreaticoduodenectomy. World J Surg. 2016;40(5):1218-25. Epub 2016/02/03. doi: 10.1007/s00268-016-3433-1. PubMed PMID: 26830906.
3. Kantor O, Talamonti MS, Sharpe S, Lutfi W, Winchester DJ, Roggin KK, et al. Laparoscopic pancreaticoduodenectomy for adenocarcinoma provides short-term oncologic outcomes and long-term overall survival rates similar to those for open pancreaticoduodenectomy. Am J Surg. 2017;213(3):512-5. Epub 2017/01/05. doi: 10.1016/j.amjsurg.2016.10.030. PubMed PMID: 28049562.
4. Liu M, Ji S, Xu W, Liu W, Qin Y, Hu Q, et al. Laparoscopic pancreaticoduodenectomy: are the best times coming? World J Surg Oncol. 2019;17(1):81. Epub 2019/05/12. doi: 10.1186/s12957-019-1624-6. PubMed PMID: 31077200; PubMed Central PMCID: PMCPMC6511193.
5. Subhas G, Mittal VK. Training minimal invasive approaches in hepatopancreatobilliary fellowship: the current status. HPB (Oxford). 2011;13(3):149-52. Epub 2011/02/12. doi: 10.1111/j.1477-2574.2010.00248.x. PubMed PMID: 21309929; PubMed Central PMCID: PMCPMC3048963.
6. Tsochatzis EA, Bosch J, Burroughs AK. Liver cirrhosis. Lancet. 2014;383(9930):1749-61. Epub 2014/02/01. doi: 10.1016/S0140-6736(14)60121-5. PubMed PMID: 24480518.
7. Befeler AS, Palmer DE, Hoffman M, Longo W, Solomon H, Di Bisceglie AM. The safety of intra-abdominal surgery in patients with cirrhosis: model for end-stage liver disease score is superior to Child-Turcotte-Pugh classification in predicting outcome. Arch Surg. 2005;140(7):650-4; discussion 5. Epub 2005/07/20. doi: 10.1001/archsurg.140.7.650. PubMed PMID: 16027329.
8. Csikesz NG, Nguyen LN, Tseng JF, Shah SA. Nationwide volume and mortality after elective surgery in cirrhotic patients. J Am Coll Surg. 2009;208(1):96-103. Epub 2009/02/21. doi: 10.1016/j.jamcollsurg.2008.09.006. PubMed PMID: 19228510.
9. Nguyen GC, Correia AJ, Thuluvath PJ. The impact of cirrhosis and portal hypertension on mortality following colorectal surgery: a nationwide, population-based study. Dis Colon Rectum. 2009;52(8):1367-74. Epub 2009/07/21. doi: 10.1007/DCR.0b013e3181a80dca. PubMed PMID: 19617746.
10. Schwartz SI. Biliary tract surgery and cirrhosis: a critical combination. Surgery. 1981;90(4):577-83. Epub 1981/10/01. PubMed PMID: 7280997.
11. Alshahrani AS, Gong GS, Yoo MW. Comparison of long-term survival and immediate postoperative liver function after laparoscopic and open distal gastrectomy for early gastric cancer patients with liver cirrhosis. Gastric Cancer. 2017;20(4):744-51. Epub 2016/12/13. doi: 10.1007/s10120-016-0675-4. PubMed PMID: 27942875; PubMed Central PMCID: PMCPMC5486494.
12. Yamamoto N, Okano K, Oshima M, Akamoto S, Fujiwara M, Tani J, et al. Laparoscopic splenectomy for patients with liver cirrhosis: Improvement of liver function in patients with Child-Pugh class B. Surgery. 2015;158(6):1538-44. Epub 2015/06/14. doi: 10.1016/j.surg.2015.05.008. PubMed PMID: 26070848.
13. Zhou S, Zhu H, Li Z, Ying X, Xu M. Safety of laparoscopic resection for colorectal cancer in patients with liver cirrhosis: A retrospective cohort study. Int J Surg. 2018;55:110-6. Epub 2018/05/31. doi: 10.1016/j.ijsu.2018.05.730. PubMed PMID: 29842931.
14. Cheung TT, Dai WC, Tsang SH, Chan AC, Chok KS, Chan SC, et al. Pure Laparoscopic Hepatectomy Versus Open Hepatectomy for Hepatocellular Carcinoma in 110 Patients With Liver Cirrhosis: A Propensity Analysis at a Single Center. Ann Surg. 2016;264(4):612-20. Epub 2016/07/20. doi:

10.1097/SLA.0000000000001848. PubMed PMID: 27433917.

15. Kim DJ, Park CH, Kim W, Jin HM, Kim JJ, Lee HH, et al. Safety of laparoscopic radical gastrectomy in gastric cancer patients with liver cirrhosis. Surg Endosc. 2017;31(10):3898-904. Epub 2017/02/17. doi: 10.1007/s00464-017-5420-5. PubMed PMID: 28205032.

16. Futagawa Y, Yanaga K, Kosuge T, Suka M, Isaji S, Hirano S, et al. Outcomes of pancreaticoduodenectomy in patients with chronic hepatic dysfunction including liver cirrhosis: results of a retrospective multicenter study by the Japanese Society of Hepato-Biliary-Pancreatic Surgery. J Hepatobiliary Pancreat Sci. 2019;26(7):310-24. Epub 2019/04/25. doi: 10.1002/jhbp.630. PubMed PMID: 31017730.

17. Warnick P, Mai I, Klein F, Andreou A, Bahra M, Neuhaus P, et al. Safety of pancreatic surgery in patients with simultaneous liver cirrhosis: a single center experience. Pancreatology. 2011;11(1):24-9. Epub 2011/02/22. doi: 10.1159/000323961. PubMed PMID: 21336005.

18. Regimbeau JM, Rebibo L, Dokmak S, Boher JM, Sauvanet A, Chopin-Laly X, et al. The short- and long-term outcomes of pancreaticoduodenectomy for cancer in Child A patients are acceptable: a patient-control study from the Surgical French Association report for pancreatic surgery. J Surg Oncol. 2015;111(6):776-83. Epub 2015/02/11. doi: 10.1002/jso.23856. PubMed PMID: 25663324.

19. El Nakeeb A, Sultan AM, Salah T, El Hemaly M, Hamdy E, Salem A, et al. Impact of cirrhosis on surgical outcome after pancreaticoduodenectomy. World J Gastroenterol. 2013;19(41):7129-37. Epub 2013/11/14. doi: 10.3748/wjg.v19.i41.7129. PubMed PMID: 24222957; PubMed Central PMCID: PMCPMC3819549.

20. Benedetto U, Head SJ, Angelini GD, Blackstone EH. Statistical primer: propensity score matching and its alternatives. Eur J Cardiothorac Surg. 2018;53(6):1112-7. Epub 2018/04/24. doi: 10.1093/ejcts/ezy167. PubMed PMID: 29684154.

21. Hurwitz EE, Simon M, Vinta SR, Zehm CF, Shabot SM, Minhajuddin A, et al. Adding Examples to the ASA-Physical Status Classification Improves Correct Assignment to Patients. Anesthesiology. 2017;126(4):614-22. Epub 2017/02/18. doi: 10.1097/ALN.0000000000001541. PubMed PMID: 28212203.

22. Granito A, Bolondi L. Non-transplant therapies for patients with hepatocellular carcinoma and Child-Pugh-Turcotte class B cirrhosis. Lancet Oncol. 2017;18(2):e101-e12. Epub 2017/02/19. doi: 10.1016/S1470-2045(16)30569-1. PubMed PMID: 28214411.

23. Northup PG, Wanamaker RC, Lee VD, Adams RB, Berg CL. Model for End-Stage Liver Disease (MELD) predicts nontransplant surgical mortality in patients with cirrhosis. Ann Surg. 2005;242(2):244-51. Epub 2005/07/26. doi: 10.1097/01.sla.0000171327.29262.e0. PubMed PMID: 16041215; PubMed Central PMCID: PMCPMC1357730.

24. Pagliaro L. MELD: the end of Child-Pugh classification? J Hepatol. 2002;36(1):141-2. Epub 2002/01/24. doi: 10.1016/s0168-8278(01)00302-6. PubMed PMID: 11804679.

25. Wang M, Zhang H, Wu Z, Zhang Z, Peng B. Laparoscopic pancreaticoduodenectomy: single-surgeon experience. Surg Endosc. 2015;29(12):3783-94. Epub 2015/03/19. doi: 10.1007/s00464-015-4154-5. PubMed PMID: 25783837.

26. Kaafarani HM, Itani KM. Classification versus valuation and grading of surgical complications. J Am Coll Surg. 2009;209(2):290-1; author reply 1-2. Epub 2009/07/28. doi: 10.1016/j.jamcollsurg.2009.05.020. PubMed PMID: 19632618.

27. Bassi C, Marchegiani G, Dervenis C, Sarr M, Abu Hilal M, Adham M, et al. The 2016 update of the International Study Group (ISGPS) definition and grading of postoperative pancreatic fistula: 11 Years

After. Surgery. 2017;161(3):584-91. Epub 2017/01/04. doi: 10.1016/j.surg.2016.11.014. PubMed PMID: 28040257.

28. Wente MN, Bassi C, Dervenis C, Fingerhut A, Gouma DJ, Izbicki JR, et al. Delayed gastric emptying (DGE) after pancreatic surgery: a suggested definition by the International Study Group of Pancreatic Surgery (ISGPS). Surgery. 2007;142(5):761-8. Epub 2007/11/06. doi: 10.1016/j.surg.2007.05.005. PubMed PMID: 17981197.

29. Wente MN, Veit JA, Bassi C, Dervenis C, Fingerhut A, Gouma DJ, et al. Postpancreatectomy hemorrhage (PPH): an International Study Group of Pancreatic Surgery (ISGPS) definition. Surgery. 2007;142(1):20-5. Epub 2007/07/17. doi: 10.1016/j.surg.2007.02.001. PubMed PMID: 17629996.

30. Sola E, Sole C, Gines P. Management of uninfected and infected ascites in cirrhosis. Liver Int. 2016;36 Suppl 1:109-15. Epub 2016/01/05. doi: 10.1111/liv.13015. PubMed PMID: 26725907.

31. Lira RPC, Antunes-Foschini R, Rocha EM. Survival analysis (Kaplan-Meier curves): a method to predict the future. Arq Bras Oftalmol. 2020;83(2):V-VII. Epub 2020/03/12. doi: 10.5935/0004-2749.20200036. PubMed PMID: 32159589.

32. Speicher PJ, Nussbaum DP, White RR, Zani S, Mosca PJ, Blazer DG, 3rd, et al. Defining the learning curve for team-based laparoscopic pancreaticoduodenectomy. Ann Surg Oncol. 2014;21(12):4014-9. Epub 2014/06/14. doi: 10.1245/s10434-014-3839-7. PubMed PMID: 24923222.

33. Delitto D, Luckhurst CM, Black BS, Beck JL, George TJ, Jr., Sarosi GA, et al. Oncologic and Perioperative Outcomes Following Selective Application of Laparoscopic Pancreaticoduodenectomy for Periampullary Malignancies. J Gastrointest Surg. 2016;20(7):1343-9. Epub 2016/05/05. doi: 10.1007/s11605-016-3136-9. PubMed PMID: 27142633; PubMed Central PMCID: PMCPMC6033586.

34. Lopez-Delgado JC, Ballus J, Esteve F, Betancur-Zambrano NL, Corral-Velez V, Manez R, et al. Outcomes of abdominal surgery in patients with liver cirrhosis. World J Gastroenterol. 2016;22(9):2657-67. Epub 2016/03/15. doi: 10.3748/wjg.v22.i9.2657. PubMed PMID: 26973406; PubMed Central PMCID: PMCPMC4777990.

35. Wang X, Cai Y, Jiang J, Peng B. Laparoscopic Pancreaticoduodenectomy: Outcomes and Experience of 550 Patients in a Single Institution. Ann Surg Oncol. 2020. Epub 2020/05/10. doi: 10.1245/s10434-020-08533-3. PubMed PMID: 32382892.

36. Xu L, Ying S, Hu J, Wang Y, Yang M, Ge T, et al. Pneumonia in patients with cirrhosis: risk factors associated with mortality and predictive value of prognostic models. Respir Res. 2018;19(1):242. Epub 2018/12/06. doi: 10.1186/s12931-018-0934-5. PubMed PMID: 30514312; PubMed Central PMCID: PMCPMC6280505.

37. Goralczyk AD, Abu-Ajaj W, Tsui TY, Bauer M, Lorf T, Ramadori G, et al. Liver transplantation in patients with liver cirrhosis and active pneumonia: an observational study. Transpl Int. 2011;24(11):1068-74. Epub 2011/09/03. doi: 10.1111/j.1432-2277.2011.01310.x. PubMed PMID: 21883503.

38. Su C, Su Y, Chou CW, Liu W, Zou J, Luo H, et al. Intravenous flurbiprofen for post-thymectomy pain relief in patients with myasthenia gravis. J Cardiothorac Surg. 2012;7:98. Epub 2012/10/02. doi: 10.1186/1749-8090-7-98. PubMed PMID: 23020939; PubMed Central PMCID: PMCPMC3493291.

Click here to access/download;Figure;Fig1.tif

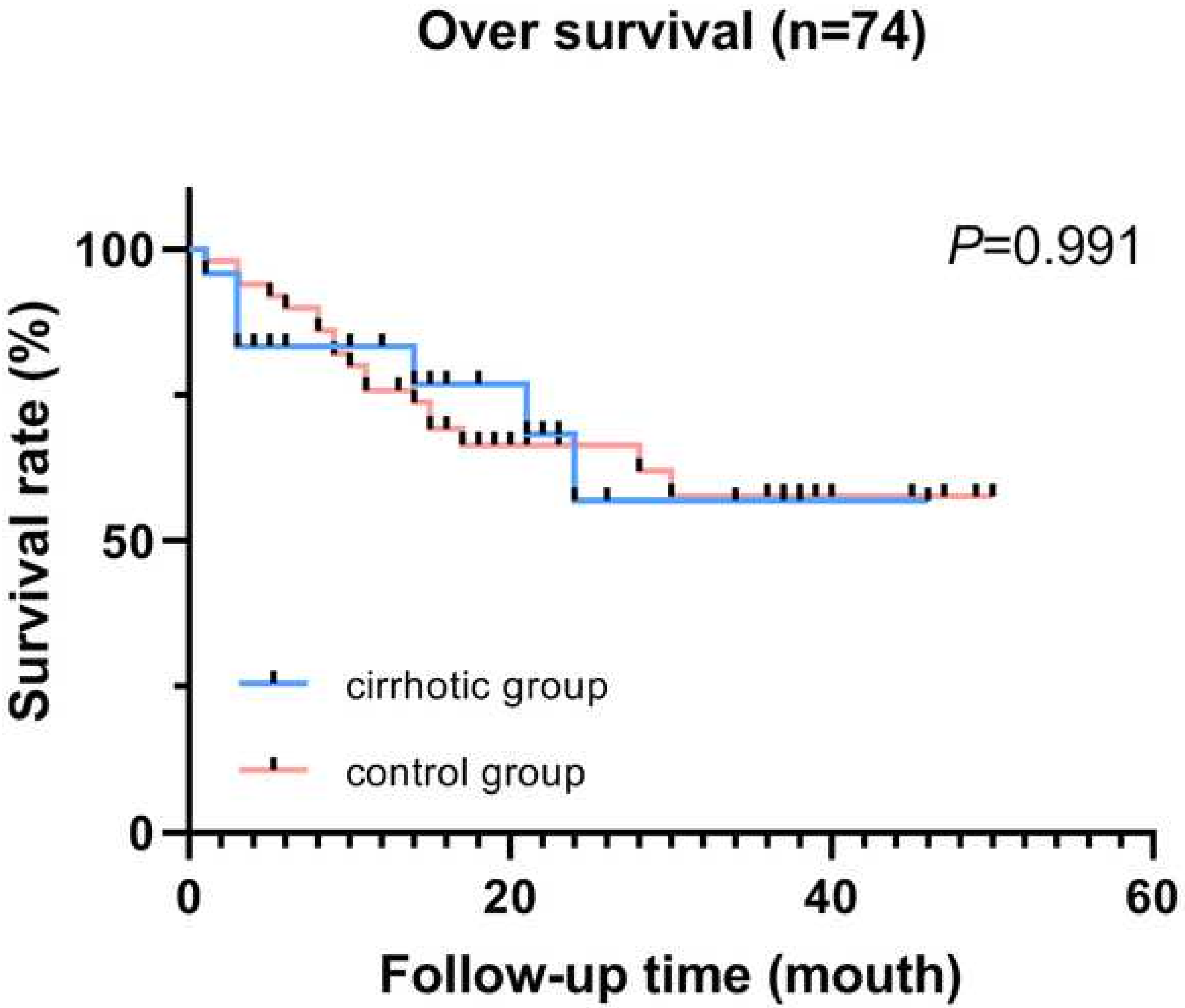

Supplement: Attachment — Submitted filename: PONE-D-20-23981_reviewer.pdf [file pone.0246364.s003.pdf]
