# Supplementary material for: Safety of laparoscopic pancreaticoduodenectomy in patients with liver cirrhosis using propensity score matching
Source: PLoS One. 2021 Jan 29;16(1):e0246364. doi: 10.1371/journal.pone.0246364 (PMC7845952; doi:10.1371/journal.pone.0246364)
Supplement: Attachment — Submitted filename: Response to Reviewers.docx [file pone.0246364.s004.docx]

Dear Prof.Chen and dear reviewers,

Re:Manuscript ID: PONE-D-20-23981 and Title: Safety of laparoscopic pancreaticoduodenectomy in patients with liver cirrhosis: a propensity score matching study

Thank you for your letter and for the reviewers’ comments concerning our manuscript entitled “Safety of laparoscopic pancreaticoduodenectomy in patients with liver cirrhosis: a propensity score matching study”. Those comments are all valuable and very helpful for revising and improving our paper, as well as the important guiding significance to our researches. We have studied comments carefully and have made correction which we hope meet with approval. Revised portion are marked in the paper. The main corrections in the paper and responds to the reviewer’s comments are as flowing:

Responds to the editor’s comments:

1. Response to comment: (Please ensure that your manuscript meets PLOS ONE's style requirements, including those for file naming.)

Response: We have revised our manuscript according to the PLOS ONE's style requirements.

2. Response to comment: (All data, which were collected in May 2020, were fully anonymized before we accessed them." Please also add this information to your ethics statement in the online submission form.)

Response: We have added it in the online submission form of ethics statement.

3. Response to comment: (We note that you have provided funding information that is not currently declared in your Funding Statement. However, funding information should not appear in the Acknowledgments section or other areas of your manuscript. We will only publish funding information present in the Funding Statement section of the online submission form.

Please remove any funding-related text from the manuscript and let us know how you would like to update your Funding Statement. Currently, your Funding Statement reads as follows:

[The author (s) received no specific funding for this work.]

Please include your amended statements within your cover letter; we will change the online submission form on your behalf.)

Response: We deleted the funding information in the acknowledgments section. We also included amended statements in our cover letter.

4. Response to comment: (Your ethics statement should only appear in the Methods section of your manuscript. If your ethics statement is written in any section besides the Methods, please delete it from any other section.)

Response: We deleted ethics statement besides the methods.

Responds to the reviewer’s comments:

Reviewer #1:

1. Response to comment:(There are a multitude of translation mistakes. I have highlighted some of them. The text should be read by a native English speaker.)

Response: We have modified the language expression in the manuscript by a language polishing service.

2. Response to comment:(On line 88 the authors say they excluded patients with severe malignant tumors? Please define severe malignant tumors.)

Response: We defined the severe malignant tumors as locally advanced, metastatic, or inoperable advanced cancer (can not be performed LPD).

3. Response to comment:(On line 148 the authors define the types of pancreatic tumors in cirrhotic group. Please define and compare these tumors with ones in the control group)

Response: We also compare these tumors in the control group (In the control group, 30 (54%) patients were diagnosed with pancreatic carcinoma, 20 (36%) hand periampullary carcinoma, 2 (4%) exhibited chronic pancreatitis whereas 4 (7%) patient had other benign or borderline pancreas tumors.).

4. Response to comment:(Also the authors do not define the types of pancreas tumors were included in the study or what stage (TNM). Please specify as some can influence the overall survival no matter what surgical technique is used. This aspect needs to be taken into account when calculating the propensity score.)

Response: In our study, all pancreatic carcinoma cases are Pancreatic ductal adenocarcinoma (PDAC). Periampullary carcinoma cases are defined as primary malignant tumors located in ampulla, duodenum, or common bile. We did not demonstrate the TNM stage and take TNM stage into account when calculating the propensity score for two reasons. Firstly, considering a relative long study period (October 2010 and December 2019), some early cases fail to record the lymphatic metastasis. Besides, the AJCC guideline revised many times in decade with changing definition in TNM stage, so we could not correctly assess the TNM stage. Secondly, the TNM stage assessment in pancreatic carcinoma and periampullary carcinoma are far different and we included many other diseases which were inapplicability in TNM stage. To sum up, we choose a more objective indicator, tumor sizes, to evaluate the tumor. Considering the complexity of different diseases, including some non-tumor cases, in our study and some cases are not applicable for TNM stage, we did not take TNM stage into calculating the propensity score. We compared the tumor sizes between cirrhotic group and control group according to different tumor. The result did not show significant difference between two groups (supplement 1).

**Supplement 1. Tumor size according to different tumor between two groups.**

|  | Patients with liver cirrhosis | Control patients | P-value |
| --- | --- | --- | --- |
| **Tumor size (cm)** |  |  |  |
| Pancreatic carcinoma (n=45) | 3.18±1.06 | 3.15±0.99 | 0.926 |
| Periampullary carcinoma （n=29） | 1.95±0.85 | 2.32±1.05 | 0.372 |

Indeed, the types of tumor can influence the overall survival. So we compare the tumor sizes between cirrhotic group and control group according to different kinds of tumor (supplement 2). The result also did not show significant difference between two groups.

**Supplement 2. Overall survival of cirrhotic patients and control patients according to different tumor.**

A: Overall survival of cirrhotic patients and control patients with pancreatic carcinoma. B: Overall survival of cirrhotic patients and control patients with periampullary carcinoma

We think the advice of reviewer is very valuable. We will standardize our medical records data collection in the future study to compensate the fault.

5. Response to comment:(Also on line 88 the authors mention that cirrhosis was defined on 3 different techniques (CT, US, biopsy). Were all of these used on every patient? Were there patients diagnosed only with ultrasound?)

Response: Not all patients were diagnosed by all methods. The cirrhotic liver was diagnosed by any one of techniques (ultrasound, CT examination, intra-operative findings, liver biopsies ).

6. Response to comment:(The authors do not mention the cause of cirrhosis. Please specify as different forms can have different impact on survival.)

Response: We have demonstrated the cause of cirrhosis in Table 2.

| **Cause of liver cirrhosis** |  |
| --- | --- |
| HBV | 13[46] |
| Alcohol abuse | 4[14] |
| Biliary-oriental cirrhosis | 7[25] |
| Schistosomiasis | 1[4] |
| Cryptogenic cirrhosis | 3[11] |

We also noticed that different forms may have different impacts on survival, but sample sizes in some types are insufficient so we could not discuss the question in the current study. We think this question is very valuable and we hope we could discuss this question in further larger sample sizes study.

Reviewer #2:

1. Response to comment:(The English language should be revised throughout the manuscript because there are mistakes and phrases that do not make sense or do not express the content well.)

Response: We have modified the language expression in the manuscript by a language polishing service.

2. Response to comment:(It should be stipulated in the manuscript whether or not the patients have signed the informed consent)

Response: We demonstrated it in the manuscript (Patients signed the informed consent that their clinic data will be used in medical study before admitted to hospital.).

3. Response to comment:(From a bibliographic point of view, the bibliographic notes should be newer than 2010 and I would suggest adding more bibliographic notes. I suggest making the stipulated changes in order to publish the manuscript later.)

Response: We have added the reference in the manuscript according to the reviewer’s note(35). Some old references aim to present previous opinions of liver cirrhosis in surgery. And we have renewed some old references(23,25,27,28).

Special thanks to you for your good comments.

Sincerely,

Dr.Peng
