# Supplementary material for: Safety of laparoscopic pancreaticoduodenectomy in patients with liver cirrhosis using propensity score matching
Source: PLoS One. 2021 Jan 29;16(1):e0246364. doi: 10.1371/journal.pone.0246364 (PMC7845952; doi:10.1371/journal.pone.0246364)
Supplement: Attachment — Submitted filename: Response to Reviewers.docx [file pone.0246364.s005.docx]

Dear Prof.Chen and dear reviewers,

Re:Manuscript ID: PONE-D-20-23981R1 and Title: Safety of laparoscopic pancreaticoduodenectomy in patients with liver cirrhosis using propensity score matching.

Thank you for your letter and for the reviewers’ comments concerning our manuscript entitled “Safety of laparoscopic pancreaticoduodenectomy in patients with liver cirrhosis using propensity score matching”. Your comments and those of the reviewers were highly insightful and enabled us to greatly improve the quality of our manuscript. In the following pages are our point-by-point responses to each of the comments of the reviewers.

Responds to the reviewer’s comments:

Reviewer #1: no response

Reviewer #3:

1. Response to comment:(In the survival data you have mentioned 25 (34%) as lost to follow up. It needs elaboration. how many of them were Cirrhotic and the remaining as non-cirrhotic matched. This is important because the cirrhotic patients are already 28, a smaller no.)

Response: We apologize for the ambiguous expressions in the manuscript. “lost during follow up” means patients died during follow up period rather than loss to follow up. We have revised the expression in the manuscript (A total of 25 (34%) patients died during follow-up. Of the 25 patients, 7 patients were in the cirrhotic group and 18 patients in the control group. ).

2. Response to comment:(This LOSS TO FOLLOW UP should be mentioned as a limitation in the discussion too.)

Response: Actually, no patient loss to follow up in our study. It is an ambiguous expression in our manuscript and we have revised it.

3. Response to comment:(The language and syntax although markedly improved, but still would require a final closer look. I noted FORM rather than the correct word FROM, 3rd line under DISCUSSION. similarly please correct CAN BE UNDERGO to CAN UNDERGO in CONCLUSIONS)

Response: We have rechecked and amended our manuscript.

4. Response to comment: (The last statement under CONCLUSIONS reads: LPD could be a safe approach for managing liver cirrhosis in patients 296 compared with PD approach., Please clarify: Do you mean you can MANAGE cirrhosis with laparoscopic pancreatico duodenectomy or you can do LPD for malignancies IN CIRRHOTIC PATIENTS?)

Response: We have revised the expression in the manuscript (LPD could be a safe approach for liver cirrhotic patients with pancreatic and periampullary diseases compared with PD approach.). We mean LPD may be a more safe approach for cirrhotic patients.

Special thanks to you for your good comments. We shall look forward to hearing from you at your earliest convenience.

Sincerely,

Dr.Peng
