# Supplementary material for: Safety of laparoscopic pancreaticoduodenectomy in patients with liver cirrhosis using propensity score matching
Source: PLoS One. 2021 Jan 29;16(1):e0246364. doi: 10.1371/journal.pone.0246364 (PMC7845952; doi:10.1371/journal.pone.0246364)
Supplement: Attachment — Submitted filename: Response to Reviewers.docx [file pone.0246364.s006.docx]

Dear Prof.Chen and dear reviewers,

Re:Manuscript ID: PONE-D-20-23981R2 and Title: Safety of laparoscopic pancreaticoduodenectomy in patients with liver cirrhosis using propensity score matching.

Thank you for your letter and for the reviewers’ comments concerning our manuscript entitled “Safety of laparoscopic pancreaticoduodenectomy in patients with liver cirrhosis using propensity score matching”. Your comments and those of the reviewers were highly insightful and enabled us to greatly improve the quality of our manuscript. In the following pages are our point-by-point responses to each of the comments of the reviewers.

Responds to the reviewer’s comments:

Reviewer #1: no response

Reviewer #3:

1. Response to comment: (What do you mean by writing"significantly different" from control group. Please explain the Fig. 1 and survival data of both groups clearly. In the Fig. the last half of the curve is the same for both the groups. )

Response: During the language polishing, the sentence was changed. We writed “no significantly different” but the “no” is missed in the last version. Sorry for the fault. Actually, the two groups have no significant difference in OS though Kaplan-Meier analysis (P=0.991).

2. Response to comment: ( In the table you have mentioned 90 mortality as 25% in cirrhotic patients and 5% in control group. That means significantly more in cirrhotic patients (expected and understandable).)

Response: We compared the 90-day mortality between cirrhotic group and control group. Although cirrhotic group have higher mortality (14%) compared with control group (5%), but the result is no significantly different (P=0.163).

|  | Patients with liver cirrhosis [%] (n=28) | Control patients [%] (n=56) | P-value |
| --- | --- | --- | --- |
| **Hospital mortality** | 1[4] | 1[2] | 0.613 |
| **90-day mortality** | 4[14] | 3[5] | 0.163 |

This is a part of our table, more details can be found in Table 4.

Special thanks to you for your good comments. We shall look forward to hearing from you at your earliest convenience.

Sincerely,

Dr.Peng
